# Effects of caffeine ingestion on dynamic visual acuity: a placebo-controlled, double-blind, balanced-crossover study in low caffeine consumers

**DOI:** 10.1007/s00213-021-05953-1

**Published:** 2021-08-22

**Authors:** Beatríz Redondo, Raimundo Jiménez, Rubén Molina, Kristine Dalton, Jesús Vera

**Affiliations:** 1 CLARO (Clinical and Laboratory Applications of Research in Optometry) Research Group, Department of Optics, Faculty of Sciences, University of Granada, Campus de la Fuentenueva 2, 18001 Granada, Spain; 2 School of Optometry & Vision Science, University of Waterloo, Waterloo, Canada

**Keywords:** Visual perception, Caffeine, Ergogenic effect, Psychostimulants, Visual function

## Abstract

**Background:**

Acute caffeine ingestion has been associated with improvements in cognitive performance and visual functioning. The main objective of this study was to determine the effects of caffeine intake on dynamic visual acuity (DVA).

**Methods:**

Twenty-one low caffeine consumers (22.5 ± 1.6 years) took part in this placebo-controlled, double-blind, and balanced crossover study. In two different days and following a random order, participants ingested either caffeine (4 mg/kg) or placebo, and DVA was measured after 60 min of ingesting the corresponding capsule. A recently developed and validated software (moV& test, V&mp Vision Suite, Waterloo, Canada) was used to assess DVA.

**Results:**

We found a greater accuracy for both the horizontal and random motion paths of DVA after caffeine ingestion (*p* < 0.001 and *p* = 0.002, respectively). In regard to the speed of the response, our data revealed that caffeine intake was associated with a faster reaction time for horizontally (*p* = 0.012) but not for randomly (*p* = 0.846) moving targets. Also, participants reported higher levels of perceived activation after consuming caffeine in comparison to placebo (*p* < 0.001).

**Conclusions:**

Our data suggest that caffeine intake (i.e., a capsule containing 4 mg/kg) has an ergogenic effect on DVA, which may be of special relevance in real-word contexts that require to accurately and rapidly detect moving targets (e.g., sports, driving, or piloting).

## Introduction

Dynamic visual acuity (DVA) is defined as the ability to resolve fine details when there is relative motion between the target and the observer (Miller and Ludvigh 1962). Research has demonstrated that DVA plays an important role in multiple real-word situations, mainly in those that involve operating in dynamic environments with moving objects such as different ball sports (Ishigaki and Miyao 1993; Quevedo-Junyent et al. 2011), driving (Hwang et al. 2020), and piloting (Kohl et al. 1991; Peters et al. 2011). DVA is considered a complex perceptual ability, which requires an appropriate target detection, peripheral awareness, oculomotor functioning, and information processing (Hoffman 1981). However, cognitive processes such as these are sensitive to different external factors such as diurnal variations (Blatter and Cajochen 2007), level of expertise (Ishigaki and Miyao 1993; Williams and Davids 1998), sleep deprivation (Krause et al. 2017), or psychostimulants (McLellan et al. 2016; Connell et al. 2017), among many other factors.

In regard to psychostimulants, caffeine is the most widely consumed psychoactive ingredient, whose popularity is attributed to its benefits for physiological, psychomotor, and cognitive performance (Glade 2010). In the last years, there has been increasing interest in the positive effects of caffeine on a number of visual skills. For example, Connell et al. (2017) observed that caffeine modulates the function of the oculomotor system, increasing the velocity of rapid eye movements. Recent scientific evidence also indicates that caffeine intake has an effect on different visual skills such as contrast sensitivity (Tsunoda et al. 2019), ocular aberrations (Bardak et al. 2016), and accommodation (Redondo et al. 2019). In addition, the ingestion of caffeine has been shown to enhance visual processing, facilitating the detection of visual stimuli and response preparation (Kenemans and Lorist 1995).

Taking into account the multiple benefits associated with caffeine consumption on cognitive performance and visual functioning, it is plausible to expect that caffeine ingestion may influence the ability to detect moving objects (i.e., DVA). Nevertheless, to the best of our knowledge, there are no studies that have assessed this hypothesis. In order to address the limitations found in the scientific literature, the aim of this study was to assess the acute effects of caffeine ingestion on DVA. To do so, DVA was measured with a recently developed and validated test (moV&; V&MP Vision Suite) (Hirano et al. 2017) before and after the ingestion of a capsule of 4 mg/kg of caffeine or placebo on two different days in a randomized order. Complementarily, we obtained the perceived levels of activation in order to check the effectiveness of caffeine/placebo manipulation.

## Methods

### Participants and ethical approval

An a priori power analysis for a three-way repeated measure ANOVA, using the GPower 3.1 software (Faul et al. 2007), was conducted to calculate the minimum sample size. We assumed an effect size of 0.30, alpha of 0.05, and power of 0.80, and it projected that seventeen participants were required for this study. After completing the power analysis, twenty-one low caffeine consumers (11 women and 10 men; mean age ± standard deviation = 22.5 ± 1.6 years; mean weight ± standard deviation = 68.4 ± 9.9 kg) took part in this study. All participants met the following inclusion criteria: (i) free of any ocular disease, as assessed by slit lamp and direct ophthalmoscopy examination; (ii) had an uncorrected refractive error lower than 0.50 D of myopia and astigmatism and 1 D of hyperopia; (iii) had a static visual acuity at far distance ≤ 0 logMAR in each eye with their best optical correction; (iv) belonged to the low visual discomfort group with the Conlon survey (Conlon et al. 1999); and (v) had no history of allergy to xantic bases or cardiovascular problems. This study adhered to the tenets of the Declaration of Helsinki and was approved the University of Granada Institutional Review Board (IRB approval: 438/CEIH/2017). All participants signed an informed consent form.

### Subjective questionnaires

In order to ensure that participants attended the laboratory under similar conditions, they reported their subjective levels of arousal before each experimental session (placebo and caffeine). Subjective levels of arousal were assessed using the Stanford Sleepiness Scale (SSS) which consists in a 7-point Likert, ranging from 1 “very active, alert or awake” to 7 “very sleepy” (Hoddes et al. 1973). Also, at the beginning of each experimental session, as well as 60 min after caffeine/placebo intake, participants were asked to complete a visual analogue scale in order to determine their subjective level of activation (0 absolutely not activated and 10 extremely activated), which has been used in similar investigations (Redondo et al. 2019, 2020).

### Dynamic visual acuity test

DVA was measured using the moV& test (V&mp Vision Suite, Waterloo, Canada), which has been validated and demonstrated good test–retest repeatability (Hirano et al. 2017). The target used was a single letter “Tumbling E” chart, which was presented in black on a white background at four orientations (right, left, up, or down). Participants, who wore their best optical correction, had to indicate the correct orientation of the branches of the letter E with the arrow keys of the keyboard. The DVA test started with size letters of 0.8 logMAR, and five letters were consecutively presented with each stimulus size. If participants correctly identified at least 3 out of 5 trials, the letter size was reduced in steps of 0.1 logMAR. The test was interrupted when participants did not identify 3 or more targets for a given letter size.

The testing was performed at 4 m using a 55-inch TV monitor (Sony 55-XF9005, Tokyo, Japan). We measured DVA for horizontal (target moved across the screen from left to right only once) and random motion paths (target moved following a random Brownian motion) at four target speeds based on the limitations of the ocular pursuit system (0.34, 0.71, 1.46, and 2.31 m/s, which is equivalent to 5, 10, 20, and 30°/s) (Gresty & Leech, 1977; Yee, 2017). Following a randomized order, each participant performed a total of 8 trials (2 motion paths × 4 target speeds). The target was shown for a maximum 20 s or until participants responded to the orientation of the letter; then, the next trial immediately started. Visual acuity (VA) and reaction time (RT) were analyzed for each trial.

### Procedure

Participants visited the laboratory on three different days, with all experimental sessions being scheduled at the same time (± 1 h) to avoid the influence of circadian variations (Read et al. 2008). In the first session, a complete visual examination was performed by an experienced optometrist to verify that subjects met inclusion criteria, as well as to obtain information about their daily caffeine consumption and their anthropometric characteristics. They were also familiarized with the software used for DVA assessment and were allowed to practice for 5 min. The second and third experimental sessions comprised the main experimental part of this study, with both sessions being identical, except for the ingestion of caffeine or placebo. Upon arrival to the laboratory, participants were asked to fill the SSS and report their level of activation and perform the DVA test. After this testing, participants ingested, in counterbalanced order, a capsule of caffeine or placebo along with a cup of water (100 ml). Sixty minutes after caffeine/placebo ingestion, participants reported their level of activation and performed the DVA test.

Each placebo capsule was comprised of 300 mg of corn starch, and the caffeine capsules (caffeine anhydrous) were dispensed in steps of 20 mg, being prepared based on participant’s weight (~ 4 mg/kg). The average weight of the experimental sample was 68.4 ± 9.9 kg, which resulted in an average caffeine dosage of 273.3 ± 40.7 mg. Both were prepared by a pharmacist laboratory (Acofarma distribución S.A., Madrid, Spain) and packaged identically in an opaque gelatine capsule to avoid identification of contents by shape, taste, or color. Aiming to accomplish the double-blind procedure, the capsules were coded and prepared by a third person.

### Statistical analyses

The normal distribution of the data (Shapiro–Wilk test) and the homogeneity of variances (Levene’s test) were confirmed (*p* > 0.05). In order to assess whether participants had similar levels of alertness/sleepiness in both experimental sessions, a paired samples *t*-test, considering the session (session 1, session 2) as the only within-participants factor, was performed for the scores reported on the SSS at the beginning of each session. Also, the impact of caffeine consumption on the levels of activation was checked by a two-way repeated measure ANOVA (substance [caffeine, placebo], point of measure [pre, 60 min]) that was carried out. For the main analyses, we performed four separate three-way repeated measures ANOVA (substance [caffeine, placebo], point of measure [pre, 60 min], and target velocity [velocity 1, velocity 2, velocity 3, velocity 4]) for horizontal RT, random RT, horizontal DVA, and random DVA. Effect sizes were reported by means of Cohen’s d and partial eta-squared (ƞ_p_^2^) for *t* and *F* tests, respectively. The level of statistical significance was set at 0.05, and the Holm-Bonferroni procedure was applied for multiple comparisons.

## Results

### Effectiveness of the experimental manipulation

**Fig. 1:**
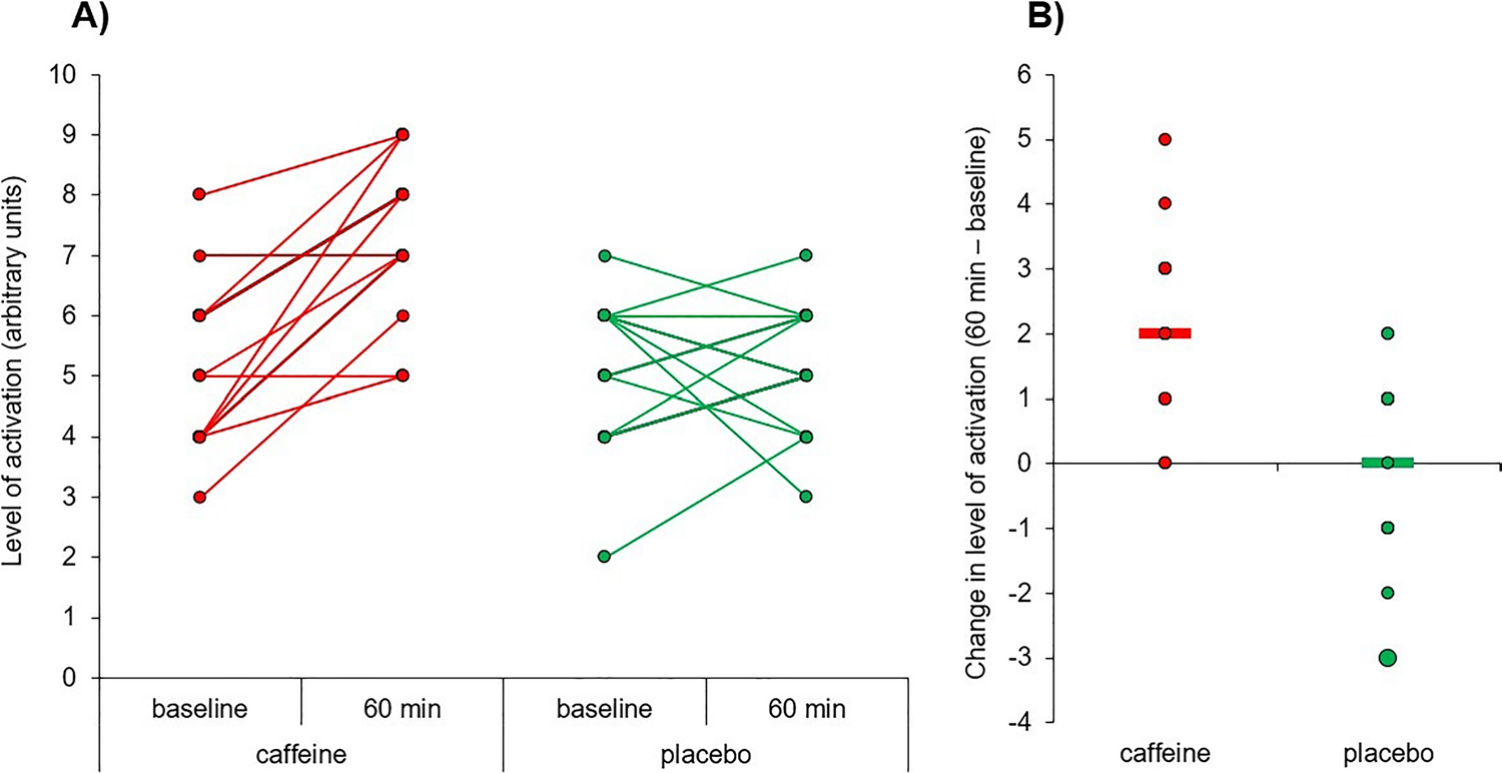
Individual comparisons of the subjective scores of activation in the caffeine and placebo conditions before and after 60 min of capsule ingestion (**A**) and scatterplot of the changes in subjective scores of activation in the caffeine and placebo conditions (**B**). The horizontal lines in panel **B** indicate the average change

Participants reported comparable levels of sleepiness/alertness at the beginning of both experimental sessions (*t*_20_ = 0.271, *p* = 0.789, *d* = 0.06). For the level of activation, there were significant differences for the substance (*F*_1,20_ = 40.51, *p* < 0.001, *ƞ*_*p*_^2^ = 0.67), point of measure (*F*_1,20_ = 39.17, *p* < 0.001, *ƞ*_*p*_^2^ = 0.66), and the interaction substance × point of measure (*F*_1,20_ = 26.36, *p* < 0.001, *ƞ*_*p*_^2^ = 0.57). Consequently, we performed two separate paired samples *t*-tests for the subjective scores of activation in both experimental sessions (caffeine, placebo), considering the point of measure as the only within-participants factor. These analyses revealed that participants reported higher levels of activation after caffeine intake (*t*_20_ = 8.22, *p* < 0.001, *d* = 1.79), but not after ingesting the placebo capsule (*t*_20_ = 0.34, *p* = 0.741, *d* = 0.08) (Fig. 1).

### Effects of caffeine intake on DVA

Table 1 shows descriptive values for the DVA parameters assessed in this study.

**Table 1:** Descriptive values (mean ± standard deviation) for the dynamic visual acuity parameters assessed at the different measurement moments in both experimental conditions

		**Velocity 1 (0.34 m/s)**		**Velocity 2 (0.71 m/s)**		**Velocity 3 (1.46 m/s)**		**Velocity 4 (2.31 m/s)**	
		*Pre*	*60 min*	*Pre*	*60 min*	*Pre*	*60 min*	*Pre*	*60 min*
**RT horizontal (ms)**	*Caffeine*	890 ± 130	832 ± 100	848 ± 125	820 ± 92	777 ± 82	745 ± 56	733 ± 114	705 ± 81
	*Placebo*	878 ± 159	865 ± 156	826 ± 113	835 ± 142	791 ± 215	790 ± 140	734 ± 128	752 ± 125
**RT random (ms)**	*Caffeine*	835 ± 111	832 ± 112	933 ± 179	916 ± 189	1040 ± 254	1018 ± 221	1117 ± 271	1057 ± 216
	*Placebo*	824 ± 97	817 ± 113	923 ± 156	922 ± 148	1022 ± 170	1015 ± 171	1136 ± 230	1129 ± 193
**VA horizontal (logMAR)**	*Caffeine*	0.07 ± 0.12	0.05 ± 0.09	0.19 ± 0.14	0.11 ± 0.09	0.33 ± 0.13	0.25 ± 0.11	0.42 ± 0.18	0.31 ± 0.16
	*Placebo*	0.05 ± 0.12	0.06 ± 0.12	0.16 ± 0.11	0.18 ± 0.14	0.31 ± 0.11	0.27 ± 0.13	0.39 ± 0.14	0.34 ± 0.13
**VA random (logMAR)**	*Caffeine*	0.15 ± 0.13	0.11 ± 0.16	0.26 ± 0.13	0.18 ± 0.14	0.39 ± 0.19	0.33 ± 0.15	0.51 ± 0.20	0.43 ± 0.17
	*Placebo*	0.15 ± 0.08	0.13 ± 0.14	0.23 ± 0.11	0.23 ± 0.14	0.40 ± 0.10	0.38 ± 0.13	0.48 ± 0.16	0.49 ± 0.16

Note: *RT* reaction time, *VA* visual acuity, *ms* milliseconds, *logMAR* logarithm of minimum angle of resolution, *m/s* meters per second

**Fig. 2:**
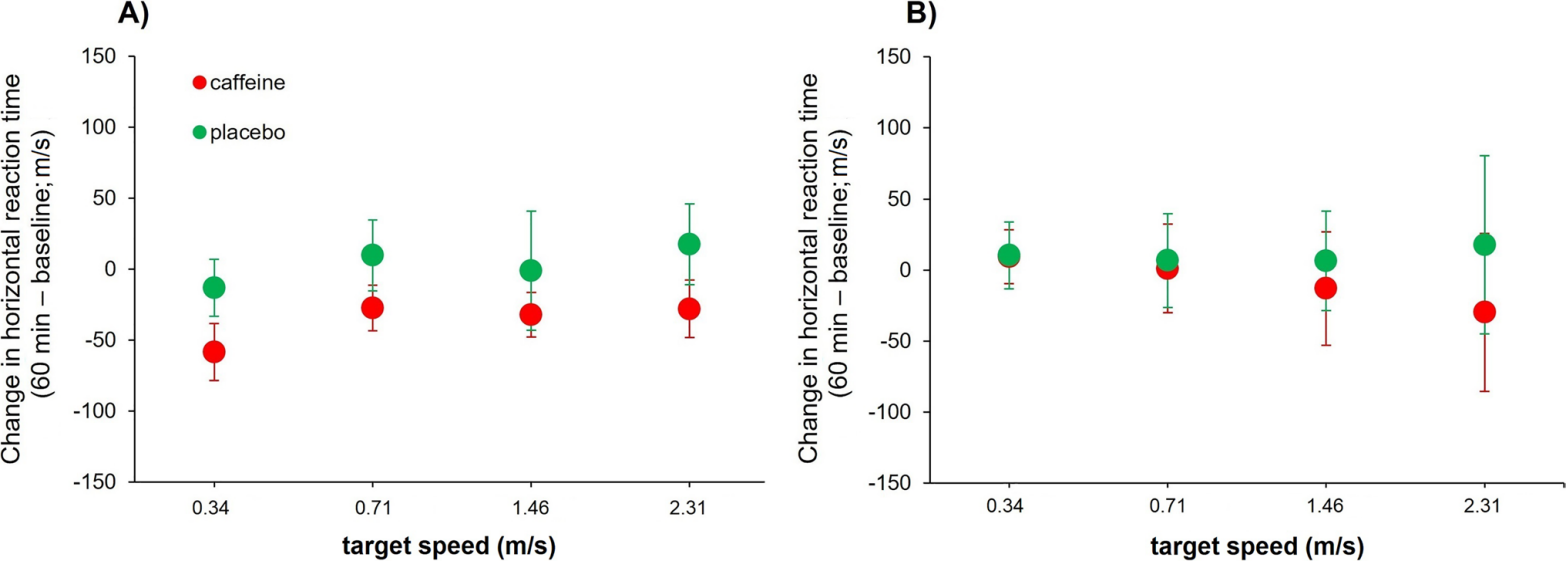
Mean changes in horizontal (**A**) and random (**B**) reaction times in the caffeine (in red) and placebo (in green) conditions at the four velocities in which the visual target was presented. Values are calculated as the difference between the measure taken after 60 min of caffeine/placebo intake and the baseline measure taken at baseline. Error bars show the standard error. All values are calculated across participants (*n* = 21). Note: ms, milliseconds; logMAR, logarithm of minimum angle of resolution; m/s, meters per second; min, minutes

For the horizontal RT, we found a statistically significant effect for the target velocity (*F*_3,60_ = 25.28, *p* < 0.001, *ƞ*_*p*_^2^ = 0.56) and the interaction “substance × point of measure” (*F*_1,20_ = 4.37, *p* = 0.049, *ƞ*_*p*_^2^ = 0.18). No differences were observed for the main effects of substance (*F*_1,20_ = 0.38, *p* = 0.546) and point of measure (*F*_1,20_ = 1.79, *p* = 0.197), as well as any other interactive effect (all Ps > 0.544). Complementarily, we performed two separate (2 [point of measure] × 4 [velocity]) ANOVAs for the caffeine and placebo conditions. For the caffeine condition, the horizontal RT was significantly shorter after caffeine intake (*F*_1,20_ = 7.64, *p* = 0.012, *ƞ*_*p*_^2^ = 0.28), and faster target velocities caused shorter horizontal RTs (*F*_3,60_ = 24.09, *p* < 0.001, *ƞ*_*p*_^2^ = 0.55). The interactive effect “point of measure × target velocity” did not reach statistical significance (*F*_3,60_ = 1.06, *p* = 0.373). For the placebo condition, faster target velocities were associated with shorter horizontal RTs (*F*_3,60_ = 9.86, *p* < 0.001, *ƞ*_*p*_^2^ = 0.33), but no effects were observed for the point of measure (*F*_1,20_ = 0.04, *p* = 0.852) or the interaction “point of measure × target velocity” (*F*_3,60_ = 0.23, *p* = 0.877) (Fig. 2A).

The analysis of the random RT yielded a statistically significant effect for the target velocity (*F*_3,60_ = 58.49, *p* < 0.001, *ƞ*_*p*_^2^ = 0.75), but no differences were obtained for the main effects of substance (*F*_1,20_ = 0.04, *p* = 0.846) and point of measure (*F*_1,20_ = 0.67, *p* = 0.424), as well as for any interaction (all Ps > 0.505) (Fig. 2B).

**Fig. 3:**
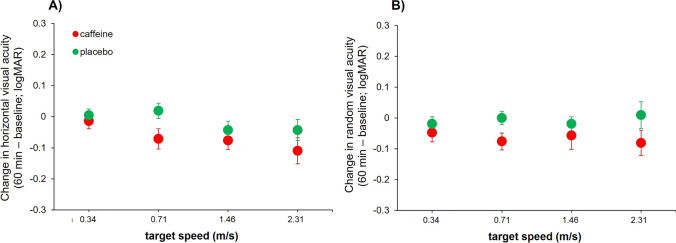
Mean changes in horizontal (**A**) and random (**B**) visual acuities in the caffeine (in red) and placebo (in green) conditions at the four velocities in which the visual target was presented. Values are calculated as the difference between the measure taken after 60 min of caffeine/placebo intake and the baseline measure taken at baseline. Error bars show the standard error. All values are calculated across participants (*n* = 21). Note: logMAR, logarithm of minimum angle of resolution; m/s, meters per second; min, minutes

For the horizontal DVA, there were a statistically significant effect of the point of measure (*F*_1,20_ = 16.17, *p* < 0.001, *ƞ*_*p*_^2^ = 0.45), target velocity (*F*_3,60_ = 117.37, *p* < 0.001, *ƞ*_*p*_^2^ = 0.85), and the interaction “substance × point of measure” (*F*_1,20_ = 6.03, *p* = 0.023, *ƞ*_*p*_^2^ = 0.23). Also, the interaction “point of measure × target velocity” (*F*_3,60_ = 2.70, *p* = 0.053, *ƞ*_*p*_^2^ = 0.12) showed a marginal effect, whereas no statistically significant differences were obtained for main effect of substance (*F*_1,20_ = 0.08, *p* = 0.785), the interactions “substance × target velocity” (*F*_3,60_ = 0.21, *p* = 0.889), and “substance × point of measure × target velocity” (*F*_3,60_ = 0.50, *p* = 0.685). Thus, we carried out separate (2 [point of measure] × 4 [velocity]) ANOVAs for the caffeine and placebo conditions in order to clarify the differences observed for the interactions in the main analysis. In the caffeine condition, we obtained a statistically significant effect for the point of measure (*F*_1,20_ = 17.16, *p* < 0.001, *ƞ*_*p*_^2^ = 0.46) and target velocity (*F*_3,60_ = 77.83, *p* < 0.001, *ƞ*_*p*_^2^ = 0.80), showing a better DVA after caffeine intake and with lower velocities of target motion. No differences were observed for the interaction “point of measure × target velocity” (*F*_3,60_ = 1.78, *p* = 0.230). For the placebo condition, the main factor of target velocity reached statistical significance (*F*_3,60_ = 67.03, *p* < 0.001, *ƞ*_*p*_^2^ = 0.77), with lower velocities being associated with better VA. However, no effects were obtained for the main effect of point of measure (*F*_1,20_ = 1.38, *p* = 0.255) or the interaction “point of measure × target velocity” (*F*_3,60_ = 1.37, *p* = 0.261) (Fig. 3A).

Lastly, we analyzed the changes in random DVA, observing a statistically significant effect for the point of measure (*F*_1,20_ = 9.05, *p* = 0.007, *ƞ*_*p*_^2^ = 0.31), target velocity (*F*_3,60_ = 124.51, *p* < 0.001, *ƞ*_*p*_^2^ = 0.86), and the interaction “substance × point of measure” (*F*_1,20_ = 6.17, *p* = 0.022, *ƞ*_*p*_^2^ = 0.24). The main effect of substance (*F*_1,20_ = 0.75, *p* = 0.398) and the rest of interactions (all Ps > 0.704) did not reach statistical significance. Again, two separate (2 [point of measure] × 4 [velocity]) ANOVAs for the caffeine and placebo conditions were conducted. For the caffeine condition, there were statistically significant differences for the point of measure (*F*_1,20_ = 12.26, *p* = 0.002, *ƞ*_*p*_^2^ = 0.3) and target velocity (*F*_3,60_ = 52.91, *p* < 0.001, *ƞ*_*p*_^2^ = 0.73), but not for the interaction “point of measure × target velocity” (*F*_3,60_ = 0.19, *p* = 0.903). Overall, the ingestion of caffeine lead to better DVA, and faster velocities of target motion were associated with worse DVA. The analysis of the placebo condition exhibited a statistically significant effect of target velocity (*F*_3,60_ = 125.13, *p* < 0.001, *ƞ*_*p*_^2^ = 0.86), indicating that faster velocities of target motion caused reduced DVAs. No differences were obtained for the point of measure (*F*_1,20_ = 0.23, *p* = 0.634) or the interaction “point of measure × target velocity” (*F*_3,60_ = 0.25, *p* = 0.862) (Fig. 3B).

## Discussion

Although there is evidence that caffeine improves several visual and cognitive skills, no studies have investigated the acute effects of caffeine on DVA performance. In view of this, we decided to investigate the changes in DVA after caffeine (~ 4 mg/kg) or placebo consumption. Our results showed a significant improvement in DVA after caffeine ingestion for both the horizontal and random motion paths. RT was also significatively shorter after caffeine intake for the horizontal motion but not for the random motion condition. As expected, we did not find significant effects for the placebo condition for DVA and RT. Faster velocities of target motion were associated with worse DVA and faster RT in both caffeine and placebo conditions. In addition, greater levels of activation were obtained in the caffeine condition, confirming the arousing effect of caffeine shown in the related literature (Glade 2010).

Caffeine is a central nervous system stimulant that acts as an adenosine receptor antagonist (Ferré 2008). There is a considerable amount of research illustrating that caffeine ingestion modifies human behavior (Lorist and Tops 2003; Smith et al. 2003; Glade 2010; Einöther and Giesbrecht 2013; McLellan et al. 2016; Connell et al. 2017; Pomportes et al. 2017). In this regard, our results showed that caffeine consumption increased subjective levels of activation, which agrees with many studies that proved that caffeine enhances alertness and feelings of wakefulness and energy (Zwyghuizen-Doorenbos et al. 1990; Smith 2002; Lorist and Tops 2003).

The ingestion of caffeine improves cognitive and psychomotor performance. For example, studies examining the acute effects of caffeine on RT have found that caffeine consumption improves the accuracy and speed responses of simple RT (Clubley et al. 1979; Jacobson and Edgley 1987; Smith et al. 1994) and choice RT (Mackay et al. 2002; Smith et al. 2003; Smith 2009; Giles et al. 2012). In the same line, we observed that caffeine reduced the time used to detect the moving objects in the DVA test. The faster RTs observed under the effects of caffeine could be explained by the positive influence of caffeine on stimulus processing and decision-making (Saville et al. 2018). However, we just observed this effect (faster RT after caffeine intake) for horizontally moving targets, but not with the random motion path, which could be due to the shorter presentation time of the horizontal targets.

Previous studies have found that DVA is dependent on target velocity, showing a negative association with target velocity (Demer and Amjadi 1993; Long and Vogel 1998). Here, we also found that DVA was worse with faster target speeds in both the caffeine and placebo conditions. Interestingly, our results showed that participants correctly identified smaller moving stimuli after caffeine ingestion compared to ingestion of the placebo. This finding was observed for both horizontally and randomly moving targets, which suggests that caffeine ingestion improved DVA. To the best of our knowledge, there are no studies that have assessed the impact of caffeine on visual acuity, in either static or dynamic conditions. However, it should be noted that while good static visual acuity is a necessary for a good DVA, the correlation between static and DVA is low (Fergenson and Suzansky 1973) because DVA is thought to be a more complex visual function task that involves additional perceptual processes (Quevedo-Junyent et al. 2011). Eye movements and contrast sensitivity, which are implicated in DVA performance, are sensitive to caffeine. Specifically, caffeine increases peak saccade velocity (Connell et al. 2017) and improves perceptual contrast sensitivity (Nguyen et al. 2018; Tsunoda et al. 2019). In addition, visual processing is more effective under caffeine effects, since it allows to improve the ability to analyze spatial frequencies orientation (Kenemans and Lorist 1995) and increase selectivity of relevant information (Lorist et al. 1996). Therefore, the effects of caffeine on DVA could result from improving any, or all, of these attributes required for an accurate DVA. However, our results cannot confirm which mechanisms explain the current findings, and further studies are required to ascertain the underlying mechanisms responsible of the benefits of caffeine intake on DVA.

DVA is considered a visual ability with high ecological validity and determines our performance in several tasks and activities of daily life (National Research Council (US) Committee on Vision 1985). In this study, we observed that caffeine ingestion improves DVA performance. Therefore, the ingestion of caffeine could be recommended in tasks that have demanding attentional requirements and/or tasks that require good resolution of moving targets such as driving or dynamic sports. The current results may be of interest in research and applied settings; however, this study presents some limitations that should be acknowledged. First, the behavioral response to caffeine depends on habitual caffeine intake (Attwood et al. 2007; Einöther and Giesbrecht 2013). Here, participants were low caffeine consumers (less than 2 cups of coffee per day), and it is plausible that the effect of caffeine intake on DVA was likely more pronounced in this experimental sample. It would be interesting to study high caffeine consumers to determine if the effect of caffeine on DVA performance is subject to tolerance. Second, DVA is related to external factors such as age, stimuli contrast, and exposure time, among others (Brown 1972; Reading 1972; Ishigaki and Miyao 1994; Altinkaynak et al. 2016). In this study, these factors were kept constant, and we did not consider how they could mediate the DVA changes caused by caffeine intake. Third, we carried out a thoughtful double-blind procedure in order to ensure that participants were unaware of the substance type ingested. However, we did not ask them to report which capsule they thought they received in each session, and it would have supported the effectiveness of the double-blind procedure. Fourth, there are considerable inter-individual differences in the physiological and behavioral responsiveness to caffeine (Nehlig 2018), and also, the impact of caffeine on humans has been shown to be dependent on the dose ingested (Quinlan et al. 2000; Chen and Parrish 2009) and the time elapsed after caffeine consumption (Magkos and Kavouras 2005). It should be noted that participants of this study ingested an average caffeine dose of 273.3 ± 40.7 mg, which is equivalent to approximately two espressos based on the findings of Crozier et al. (2012) who obtained a median value of 140 mg of caffeine in servings of espresso coffee. Lastly, we used a specific psychostimulant (i.e., caffeine), and the impact of other substances on DVA has not investigated. Therefore, our findings should be cautiously interpreted in this regard, and future studies should consider assessing the mediating role of the aforementioned factors on DVA performance.

## Conclusions

This placebo-controlled, double-blind, balanced crossover study demonstrated that acute caffeine ingestion (4 mg/kg) improves dynamic visual acuity and reduces RT in comparison to placebo consumption. The positive changes of caffeine on visual and cognitive skills are proposed as a potential explanation of these findings. The acute improvements of DVA with caffeine could have positive implications in real-life activities that require a good DVA for moving targets (e.g., sports, driving, or piloting).
